# Intermittent Theta-Burst Stimulation Reverses the After-Effects of Contralateral Virtual Lesion on the Suprahyoid Muscle Cortex: Evidence From Dynamic Functional Connectivity Analysis

**DOI:** 10.3389/fnins.2019.00309

**Published:** 2019-04-24

**Authors:** Guoqin Zhang, Xiuhang Ruan, Yuting Li, E Li, Cuihua Gao, Yanli Liu, Lisheng Jiang, Lingling Liu, Xin Chen, Shaode Yu, Xinqing Jiang, Guangqing Xu, Yue Lan, Xinhua Wei

**Affiliations:** ^1^Department of Radiology, Guangzhou First People’s Hospital, Guangzhou Medical University, Guangzhou, China; ^2^The Second Affiliated Hospital, South China University of Technology, Guangzhou, China; ^3^Department of Rehabilitation Medicine, Guangzhou First People’s Hospital, Guangzhou Medical University, Guangzhou, China; ^4^Shenzhen Institutes of Advanced Technology, Chinese Academy of Sciences, Shenzhen, China; ^5^Department of Rehabilitation Medicine, Beijing Tiantan Hospital, Capital Medical University, Beijing, China

**Keywords:** repetitive transcranial magnetic stimulation, theta-burst stimulation, magnetic resonance imaging, swallowing, dynamic functional connectivity analysis

## Abstract

Contralateral intermittent theta burst stimulation (iTBS) can potentially improve swallowing disorders with unilateral lesion of the swallowing cortex. However, the after-effects of iTBS on brain excitability remain largely unknown. Here, we investigated the alterations of temporal dynamics of inter-regional connectivity induced by iTBS following continuous TBS (cTBS) in the contralateral suprahyoid muscle cortex. A total of 20 right-handed healthy subjects underwent cTBS over the left suprahyoid muscle motor cortex and then immediately afterward, iTBS was applied to the contralateral homologous area. All of the subjects underwent resting-state functional magnetic resonance imaging (Rs-fMRI) pre- and post-TBS implemented on a different day. We compared the static and dynamic functional connectivity (FC) between the post-TBS and the baseline. The whole-cortical time series and a sliding-window correlation approach were used to quantify the dynamic characteristics of FC. Compared with the baseline, for static FC measurement, increased FC was found in the precuneus (BA 19), left fusiform gyrus (BA 37), and right pre/post-central gyrus (BA 4/3), and decreased FC was observed in the posterior cingulate gyrus (PCC) (BA 29) and left inferior parietal lobule (BA 39). However, in the dynamic FC analysis, post-TBS showed reduced FC in the left angular and PCC in the early windows, and in the following windows, increased FC in multiple cortical areas including bilateral pre- and postcentral gyri and paracentral lobule and non-sensorimotor areas including the prefrontal, temporal and occipital gyrus, and brain stem. Our results indicate that iTBS reverses the aftereffects induced by cTBS on the contralateral suprahyoid muscle cortex. Dynamic FC analysis displayed a different pattern of alteration compared with the static FC approach in brain excitability induced by TBS. Our results provide novel evidence for us in understanding the topographical and temporal aftereffects linked to brain excitability induced by different TBS protocols and might be valuable information for their application in the rehabilitation of deglutition.

## Introduction

Swallowing disorders (i.e., dysphagia) are a common sequela of a range of diseases and disorders (González-Fernández and Daniels, 2008; Clave and Shaker, 2015). Patients with dysphasia are at risk of developing severe complications such as aspiration pneumonia, malnutrition, and dehydration, which have large impacts on the quality of life of patients (Martino et al., 2005). Despite various strategies that have been developed that aim to enhance swallowing recovery, efficient treatment options for dysphagia rehabilitation remain limited (Bath et al., 2000; Geeganage et al., 2012).

Transcranial magnetic stimulation (TMS) is a non-invasive method that can alter human cortex excitability and has started to attract attention for use in treating dysphagia. Increasing evidence has indicated that TMS may be a promising tool to facilitate neural reorganization in dysphagic patients (Khedr and Abo-Elfetoh, 2010; Michou et al., 2012; Doeltgen et al., 2015). TMS can modify the excitability at the site of stimulation as well as in remote brain areas that are functionally connected with the target site (brain network effect) (Suppa et al., 2008; Valchev et al., 2015; Steel et al., 2016). More recently, theta burst stimulation (TBS), a novel pattern of rTMS, was shown to produce significant and long-lasting aftereffects within very short stimulation periods (Huang et al., 2005).

There are two forms of TBS according to the stimulus pattern: intermittent TBS (iTBS) enhances cortical excitability, and in contrast, continuous TBS (cTBS) suppresses cortical excitability, which is usually explained by its long-term depression (LTD) of synaptic activity (Huang et al., 2005). The recent development of human temporary “virtual lesion” by using non-invasive magnetic stimulation at a low frequency has given us the opportunity to explore the cortical central mechanisms in improving swallowing function and recovery in a controlled environment before proceeding to clinical trials (Mistry et al., 2007; Cugy et al., 2016). Thus, as an entity for suppression of cortical excitability, cTBS can be used to create a “virtual lesion” in the swallowing cortex by transiently reducing the excitability of the targeted brain area.

It has been shown that swallowing is represented in multiple cortical regions bilaterally and swallowing functions are organized by a distributed brain network (Ertekin and Aydogdu, 2003). Although stimulating the lesioned or unlesioned hemisphere for dysphagia patients remains a controversial topic, converging evidence suggests unaffected hemisphere stimulation with TMS helps to rehabilitate swallowing function (Hamdy et al., 1998; Jefferson et al., 2009; Pisegna et al., 2016). Furthermore, our recent study indicated that iTBS could reverse the inhibitory effect induced by cTBS in the contralateral suprahyoid motor cortex (Lin et al., 2017). Most of the previous studies explored the aftereffect of TBS over the cortex by measuring the motor-evoked potentials (MEP) of peripheral muscles (Wu et al., 2012; Lin et al., 2017). However, our understanding of the underlying mechanisms linked to the neural networks between the two hemispheres of the swallowing cortex after TBS stimulation remains unclear.

Recently, resting-state functional MRI (Rs-fMRI) has been used in the investigation of the neural mechanism of TMS (Andoh et al., 2015; Bharath et al., 2015; Dichter et al., 2015; Valchev et al., 2015; Ji et al., 2017). The functional connectivity (FC) analysis calculating the temporal similarities was broadly used in Rs-fMRI studies; however, most of the studies only considered FC measurements using static FC (Biswal et al., 1995; Geerligs et al., 2014). It measures the correlations of signals between different brain regions during a scanning period, thus providing a relatively static pattern of brain activity coherence in the resting state (Wig et al., 2011). More recently, emerging evidence has proposed that human brain connectivity is most likely time-dependent and dynamic (i.e., dynamic FC) (Liu et al., 2017; Marusak et al., 2017; Robinson et al., 2017). Therefore, a dynamic FC analysis is a sensitive method to capture the time-varying information of FC and has been attracting increasing attention for use in characterizing the brain’s intrinsic functional organization (Marusak et al., 2016; Preti et al., 2016). However, as far as we know, there is no previous study that explored the features of dynamic FC of TBS on the swallowing cortex to date.

The suprahyoid muscle is linked to the movement of the hyoid-throat complex and plays an important role in swallowing (Nam et al., 2013). The purpose of this study was to evaluate dynamic functional network connectivity in subjects given iTBS following contralateral cTBS, which acts to create a “virtual lesion” on the suprahyoid muscle cortex. Although stimulating the lesioned or unlesioned hemisphere for dysphagia patients remains a controversial topic, converging evidence suggests unaffected hemisphere stimulation with TMS helps to rehabilitate swallowing function (Hamdy et al., 1998; Jefferson et al., 2009; Pisegna et al., 2016). Furthermore, a recent study indicated that iTBS could reverse the inhibitory effect induced by cTBS in the contralateral suprahyoid motor cortex (Lin et al., 2017). Therefore, to verify if the contralateral iTBS can reverse the effect of cTBS which was used as a virtual lesion on the left side, we placed the iTBS on the right motor areas of the suprahyoid muscles. In this study, we hypothesized that iTBS might facilitate bilateral brain excitability though interhemispheric interactions between the suprahyoid motor cortices and reverse the aftereffects of cTBS in the contralateral hemisphere. To verify this hypothesis, this study aimed to characterize the temporal dynamic features of whole-brain FC at a voxel level, to explore whether dynamic features of FC are linked to the underlying mechanism of iTBS in the treatment of a contralateral virtual lesion in the swallowing cortex.

## Materials and Methods

### Participants

A total of 20 participants (10 women; mean age: 23.5 ± 4.4 years) took part in the study after giving written informed consent. We did not include subjects with a history of neurological and/or psychiatric disease, swallowing dysfunction, or substance abuse. The present study was approved by the clinical research ethics committee of Guangzhou First People’s Hospital. The study was conducted in accordance with the Declaration of Helsinki (2008 revision).

### Transcranial Magnetic Stimulation

The details of the TMS procedure have been described in a previous study (Lin et al., 2017; Ruan et al., 2017). In brief, motor evoked potentials (MEPs) to TMS were recorded from the suprahyoid muscle and right first dorsal interosseous (FDI) of the hand. Electrodes (Yiruide, Wuhan, China) were placed over the bilateral suprahyoid muscle surface and the surface of the FDI to detect the suprahyoid muscle electromyographic (EMG) responses. Electrodes were connected to an EMG recording system (Yiruide, Wuhan, China) with a preamplifier and amplifier.

Magnetic stimulation was performed using a handheld figure of eight coil (mean 70 mm outer diameter) connected to a Magstim super rapid stimulator (Yiruide Medical Equipment Co., Wuhan, China) to deliver single-pulse TMS. The stimulating coil was held tangentially to the skull with the coil handle pointing backward and laterally 45° away from the anterior-posterior axis (Mistry et al., 2007). The precise position of the TMS coil was tracked and recorded using the neuronavigation system (SofTaxic, E.M.S., Bologna, Italy) with a graphic user interface and a three dimensional (3D) optical digitizer (NDI, Polaris Vicra, ON, Canada).

rTMS was delivered using the theta burst stimulation (TBS) protocol as first described by Huang et al. (2005). Briefly, the coil was held in an identical orientation to single pulse TMS when performing TBS. The navigation system was used to determine the hot spot and active motor threshold (AMT) of FDI. The motor hot spot was determined as the site at which TMS consistently elicited the largest MEPs from the contralateral FDI. Then the stimulation intensity was gradually reduced until at least 5 times of 10 consecutive stimulations which induced MEP of ≥50 μV in the contralateral thumb abductor muscle, and the stimulation intensity was the ATM of the subject. After that, the navigation system was used to determine the hot spot of the suprahyoid motor cortex. The method was similar to determination of the FDI hot spot. TBS was applied over the hot spot of the suprahyoid motor cortex. The stimulation intensity was set to 80% of the AMT of the FDI.

The TBS protocol consisted of three pulses of stimulation delivered at 50 Hz and repeated at 5 Hz. In the iTBS pattern, a 2 s train of TBS was repeated every 10 s for a total of 190 s (600 pulses in total). By contrast, a 40 s train of uninterrupted TBS was administered for approximately 40 s (600 pulses in total) in the cTBS protocol. The stimulation intensity was set to 80% of the AMT of the FDI.

### Experimental Design

**FIGURE 1:**
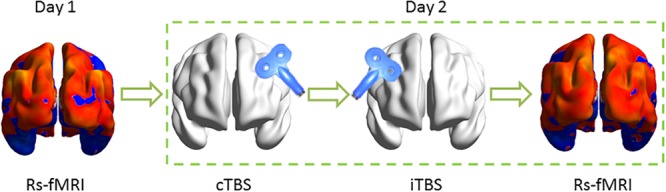
Schematic of the experimental design. Resting-state functional MRI (Rs-fMRI) was acquired before and after theta burst stimulation (TBS) on the different day. In TBS stimulation, each subject received continuous TBS (cTBS) first on the left suprahyoid muscle cortex and immediately followed intermittent TBS (iTBS) on the right homologous area. The target area for TBS stimulation was guided by neuronavigation.

The stimulation protocol was as follows: iTBS was performed on the right hemisphere immediately after completing cTBS on the left motor cortex of the suprahyoid muscles. Rs-fMRI datasets were acquired before and after the TBS implement on a different day (Figure 1).

### MR Data Acquisition

Brain imaging was performed on a 3.0 T MRI scanner (Siemens, Erlangen, Germany) using a 16-channel phased-array head coil. The Rs-fMRI data were acquired before and immediately after each TBS session. The acquisition parameters for functional data were as follows: TR = 2000 ms, TE = 21 ms, FA = 90°, FOV = 240 × 240 mm, matrix = 64 × 64, slice thickness = 4.0 mm, and voxel size = 3.75 × 3.75 × 4.0 mm. During the Rs-fMRI scan, participants were instructed to relax with their eyes closed but not fall asleep. T1-weighted structural images were acquired using a three-dimensional magnetization-prepared rapid acquisition gradient-echo (MPRAGE) sequence (TR = 2530 ms, TE = 2.93 ms, FA = 7°, FOV = 256 mm, a 256 × 256 matrix and a slice thickness of 1.0 mm).

### MR Data Preprocessing

Image preprocessing was carried out using the Data Processing Assistant for Rs-fMRI (DPARSF^1^) (Yan and Zang, 2010), which runs with the statistical parametric mapping software (SPM8^2^). Briefly, data pre-processing steps included removal of the first 10 image frames for signal equilibration; slice timing correction for acquisition delay between slices; realignment of the data to compensate for rigid body motion (excessive motion was defined as translation or rotation >2 mm or 2°); registration of the 3D structural images into the standard Montreal Neurological Institute (MNI) space; regression of white matter nuisance signals, cerebral spinal fluid BOLD-signal and 24 head-motion profiles to minimize the effect of head motion; registration of the functional images to the MNI space using the parameters of structural image normalization and with resampling to 3 × 3 × 3 mm^3^; spatial smoothing using a Gaussian kernel of 4 mm full-width at half maximum (FWHM); and bandpass filtering (0.01–0.08 Hz) of the functional data to reduce the effects of low-frequency drift and high-frequency noise (Biswal et al., 1995).

### Static Functional Connectivity Analysis

For each individual data set, we first calculated the static FC with Pearson correlation analysis. The FC was performed using REST V1.8 package^3^. Pearson’s correlation coefficients were computed between the time series of all pairs of brain voxels. Each voxel represents a node in the graph, and each significant functional connection (i.e., Pearson correlation) between any pair of voxels is an edge. As a result, we can obtain an n × n matrix of Pearson’s correlation coefficients between any pair of voxels so that the whole brain FC matrix for each participant was constructed. Then, individual correlation matrices were transformed into a Z-score matrix using Fisher’s r-to-z transformation to improve normality. After that, the weighted degree centrality strength of a voxel as the sum of the connections (*Z*-values) between a given brain voxel and all other voxels was computed. As previously described similarly (Gao et al., 2016), we used a Pearson’s correlation coefficient threshold at *r* > 0.25 by thresholding each correlation at *p* ≤ 0.001.

### Dynamic Functional Connectivity Construction

A voxel-to-voxel based dynamic functional connectivity (dynamic FC) construction was performed using the dynamic brain connectivity (dynamic BC) toolbox (Liao et al., 2014a). A time-varying parameter regression equation was employed to describe the dynamic interactions between brain regions. We calculated correlation maps using the Pearson correlation strategy between the time series derived from each voxel to all other brain voxels for a sliding window of 50 volumes (100 s). We obtained a correlation map for each sliding window; then, the correlation map was converted to z-scores using the Fisher r-to-z transformation. The window was then shifted by 0.6 volume (1.2 s) and a new correlation map was calculated. This approach permitted the estimation of FC over time (Liao et al., 2014a,b). Since the time series were composed of 200 volumes, this procedure yielded sliding-time windows and thus resulted in 121 correlation maps.

### Statistical Analysis

A paired *t*-test was performed to investigate the difference between the post-TBS and pre-TBS conditions. A threshold of *p* < 0.05, corrected using Monte Carlo simulations in the AFNI AlphaSim program^4^, was used to calculate the probability of false positive detection while accounting for both the individual voxel probability thresholding and cluster size (single voxel *p* = 0.05, FWHM = 6 mm). Using this program, clusters of greater than 85 voxels were applied to the resulting statistical map at a corrected significance level of *p* < 0.05 (Song et al., 2011; Wang et al., 2012).

## Results

### Group Differences in Static Functional Network Connectivity

**FIGURE 2:**
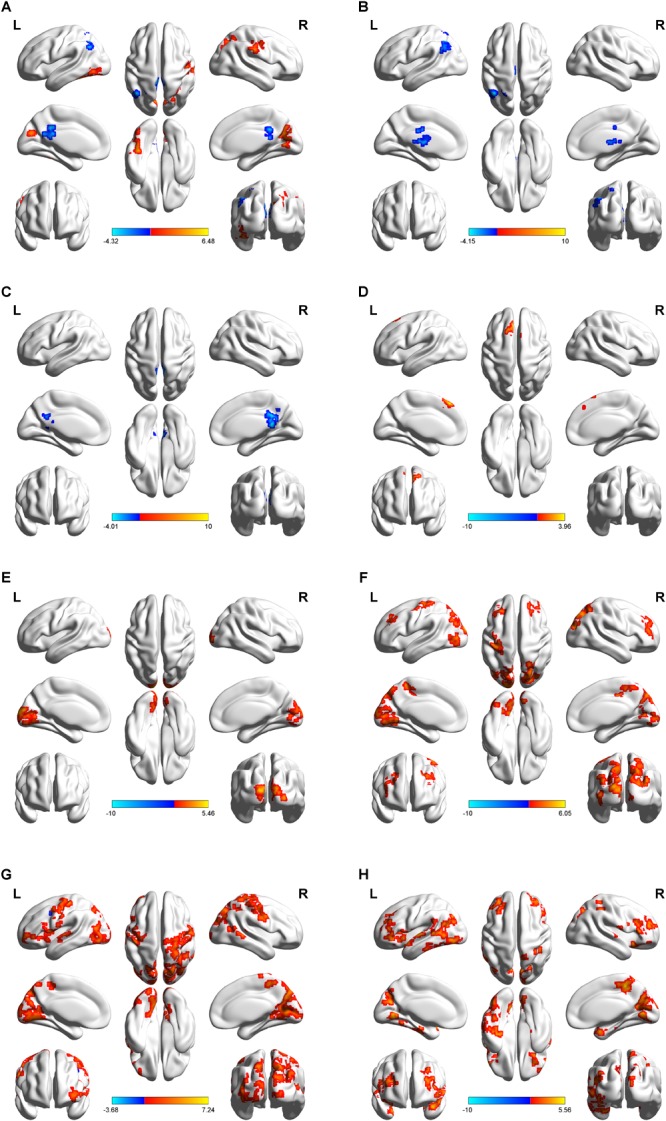
Regional distribution of altered functional connectivity between post-theta-burst stimulation and baseline in healthy subjects. Short-term of aftereffects on brain functional connectivity (FC) induced by intermittent theta burst stimulation (iTBS) followed the contralateral continuous theta burst stimulation (cTBS) (i.e. “virtual lesion”) over the suprahyoid muscle cortex. The upper images represent static FC **(A)** and a serial of dynamic FC maps **(B–H)**. Lateral, dorsal, medial, basal and posterior views are shown in every figure. Color bar indicates the *T*-value at the bottom. Areas color-coded in red (blue) indicate the regions in which the values of FC corresponding to post-TBS were higher (lower) than those of pre-TBS (*p* < 0.05, AlphaSim correction was performed to correct for multiple comparisons). Surface visualization of regions with abnormal FCs using BrainNet Viewer (http://www.nitrc.org/projects/bnv/). For static FC comparison **(A)**, increased FC was found in the precuneus, left fusiform gyrus, and right pre/post-central gyrus, and decreased FC was observed in posterior cingulate gyrus (PCC) and left inferior parietal lobule. In dynamic FC analysis, post-TBS showed reduced FC in left angular and PCC on the early windows **(B,C)**, in the following windows **(D–H)**, increased FC in multiple cortical areas including bilateral pre- and postcentral gyri and paracentral lobule, and non-sensorimotor areas including a body of prefrontal, temporal and occipital gyrus, and brain stem. The details are presented in Table 1.

As shown in Figure 2A and Table 1, compared to baseline, post-combined cTBS/iTBS exhibited increased FC in the precuneus (BA 19), left fusiform gyrus (BA 37), and right pre/post-central gyrus (BA 4/3) and showed decreased FC in the posterior cingulate gyrus (PCC) (BA 29) and left inferior parietal lobule (BA 39).

**Table 1:** Functional connectivity comparison between post- and pre-TBS on the suprahyoid muscle cortex.

Brain region	BA	Cluster size (voxels)	Peak MNI coordinates (mm)			*T*-value
			X	Y	Z	
**Static functional connectivity**					
Right pre/postcentral gyrus	4/3	87	56	–19	39	3.1934
Precuneus	19	306	24	–75	36	4.5864
Left fusiform gyrus	37	120	–39	–45	–24	6.4808
Posterior cingulate gyrus	29	144	3	–42	15	–4.1386
Left inferior parietal lobule	39	104	–42	–63	48	–4.3236
**Dynamic functional connectivity**					
**Window 1**					
Left thalamus		92	–3	–12	9	–3.9233
Left inferior parietal lobule	40	123	–42	–60	48	–4.1469
**Window 2**					
Posterior cingulate		153	12	–39	12	–4.0102
**window 3**					
Left medial superior frontal gyrus	8	87	–9	33	51	3.9604
**Window 4**					
Middle occipital gyrus/cuneus	18	448	30	–99	0	5.4572
**Window 5**					
Left precentral gyrus	6	102	–42	0	60	3.7038
Paracentral lobule	5	201	0	–39	51	4.1413
Left cuneus/precuneus	18	795	–21	–93	15	6.0536
Right precuneus	7	377	21	–78	39	4.9914
Right superior frontal gyrus	9	184	24	30	36	3.4375
Left superior frontal gyrus	9	113	–39	36	36	3.7198
Right superior temporal gyrus	39	92	48	–54	15	4.5806
**Window 6**					
Right postcentral/precentral gyrus	6/4	441	45	–54	60	5.0194
Left postcentral/precentral gyrus	6/4	248	–42	–24	63	4.6705
Precuneus/cuneus	7	1239	–18	–72	45	4.9855
Left inferior frontal operculum	45	245	–42	21	18	4.6498
Left middle occipital gyrus	19	218	–6	–105	6	7.2442
**Window 7**					
Paracentral lobule/postcentral gyrus	5	252	12	–39	54	5.0432
Precuneus/cuneus	18	375	–12	–66	–3	4.4948
Right middle frontal gyrus	10	348	57	27	30	5.0111
Right inferior frontal gyrus	38	163	27	18	–24	4.9059
Left inferior frontal gyrus	45	258	–54	36	3	5.2874
Left superior temporal gyrus	38	272	–39	15	–21	5.5615
Left middle temporal gyrus	22	401	–57	–42	6	4.6347
Left fusiform gyrus	37	855	–42	–48	–24	502446
Brainstem	NS	85	–6	–24	–33	403195

*TBS, continuous theta-burst stimulation; BA, Brodman’s area; MNI, Montreal Neurological Institute; cTBS, continuous TBS; iTBS, intermittent TBS.*

### Group Comparison of Dynamic Functional Connectivity

After dynamic FC analysis, we obtained a seven-unit dataset, and from each unit of the dataset, we calculated an FC map. Thus, a total of seven FC *T*-test maps were obtained. Compared with the pre-TBS condition, in the first window, the post-TBS exhibited decreased FC in the left inferior parietal lobule and left thalamus; in the second window, the PCC showed decreased FC. The left medial superior frontal gyrus displayed increased FC in the third window; the middle occipital gyrus/cuneus demonstrated increased FC in the fourth window; then, in the following windows, multifocal bilateral areas included sensorimotor areas, such as the primary sensorimotor cortex and paracentral lobule, and non-sensorimotor areas, such as the occipital gyrus, prefrontal gyrus, temporal gyrus and brain stem (Figure 2B–H, details in Table 1).

## Discussion

In this study, we explored the short-term after-effect of iTBS application to the right motor cortex of the suprahyoid muscles following cTBS, which was used to create a “virtual lesion” in the left suprahyoid muscles cortex in healthy participants. The static and dynamic FC analyses based on the data of Rs-fMRI were implemented to investigate the FC of the brain network at the voxel level for the whole brain. In the static FC measurement, increased FC was found in the precuneus, left fusiform gyrus, and right pre/postcentral gyrus; and decreased FC was observed in the PCC and left inferior parietal lobule. Compared with static FC, the dynamic FC displayed a different distribution of brain FC in the brain networks. From the dynamic FC maps, increased FC in multiple bilateral brain areas including bilateral sensorimotor areas and other non-sensorimotor brain regions were found, while they were not fully displayed in the static FC analysis. The present study, to our knowledge, is the first study to focus on the short-term after-effects of TBS on the swallowing cortex by examining brain network temporal dynamics in a cohort of healthy controls.

Similar to previous studies (Michou et al., 2016), cTBS, an inhibitory TMS, was applied to the left suprahyoid muscles motor area to act as a “virtual lesion,” which temporarily and reversibly disrupted a focal region of brain excitability. In contrast, iTBS was used as an excitatory stimulation on the right suprahyoid muscles motor areas in order to verify whether iTBS could alter the brain excitability after forming the contralateral “virtual lesion” and to identify what pattern of alteration of FC could be induced in the whole brain network. As expected, a number of spatially discrete cortical regions exhibited alternation of FC after the TBS application in both static FC and dynamic FC maps.

In the static FC maps, because the right motor cortex of the suprahyoid muscles was the target area for iTBS, the right primary somatosensory cortex displaying an increased FC is not surprising. A body of studies has indicated that the primary somatosensory cortex is implicated in reflexive and volitional swallowing (Hamdy et al., 1999a,b; Ertekin and Aydogdu, 2003; Martin et al., 2007) and plays a role in the initiation of the pharyngeal stage of swallowing (Malandraki et al., 2009). Additionally, the left posterior parietal cortex and PCC exhibited decreased FC in the static FC maps. Similar to the previous study (Ertekin and Aydogdu, 2003), the posterior parietal cortex together with the somatosensory cortex is likely to have a sensory role in the control of swallowing. The precuneus and PCC are components of the default-mode network (DMN) that occurs during the initiation of task-related activities (Raichle et al., 2001). Furthermore, the precuneus is thought to play a role in adjusting volitional swallowing related to swallow-related intent and planning and possibly urges (Hamdy et al., 1999a; Kern M.K. et al., 2001). Moreover, the PCC and precuneus are regarded as association areas, which are thought to play a role in integrating sensory information (Hamdy et al., 1999a). Consistent with a previous report (Jefferson et al., 2009), we did not observe decreased or increased FC in the left sensorimotor cortex, which is the target position of cTBS, and we speculate there is a balance between bilateral hemispheres in brain excitability due to the interaction between iTBS and cTBS over the opposite swallowing motor cortex. Taken together, in the static FC maps, we did not observe the evidence that indicates that iTBS can reverse the aftereffect of the contralateral “virtual lesion” induced by cTBS.

In the dynamic FC maps, we observed alterations in FC induced by TBS in multifocal and bilateral brain regions in different windows. Interestingly, some areas demonstrated in the static FC maps with alteration in FC, such as the left posterior parietal lobule and PCC, also exhibited decreased FC in the first few windows of the dynamic FC maps. Similar to a previous study (Babaei et al., 2013), the lateral parietal, PCC and precuneus regions have close FC among themselves and may be involved in sensory processing in the swallowing brain network. The left thalamus displayed decreased FC in the dynamic FC maps in the present study. It has been proposed that the thalamus is a structure known as a relay station for sensory information traveling into higher cortical areas (Malandraki et al., 2009); and thus, after the ipsilateral swallowing cortex is suppressed, the processing and transferring of sensorimotor information is inhibited accordingly. Apart from the areas with decreased FC on the dynamic FC maps, we found increased dynamic FC in the bilateral primary sensorimotor cortex in the following windows after TBS application. As we mentioned above, the activation of the primary sensorimotor during volitional swallowing has been well documented with imaging studies in the fMRI studies (Hamdy et al., 1999a,b; Mosier et al., 1999; Mihai et al., 2014). In our study, the targeted brain areas were the cortex of the bilateral suprahyoid muscle, and thus, the presence of alterations of brain connectivity in the post- and precentral gyrus is not surprising. These areas are supposed to play an important role in the initiation and regulation of swallowing (Narita et al., 1999; Ertekin and Aydogdu, 2003). In addition, the paracentral lobule and the continuation of the precentral and postcentral gyri displayed increased FC in the dynamic FC maps. The paracentral lobule is associated with sensorimotor functioning and is activated by swallowing tasks (Martin et al., 2007). Except for the sensorimotor brain regions we mentioned above, a number of other non-sensorimotor brain regions displayed alteration in FC in the present study, including the bilateral temporal lobe, frontal cortex and occipital cortex. Swallowing is a complex and dynamic neuromuscular task requiring rapid and precise coordination of numerous cranial nerves and muscle pairs (Ertekin and Aydogdu, 2003). It was supposed that the non-sensorimotor areas may represent swallowing related to intent, planning, decision making, and memory, as well as information processing related to deglutition (Ertekin and Aydogdu, 2003). Specifically, the temporal cortex, together with the prefrontal cortex, is supposed to play a supplementary role in the regulation of swallowing and feeding because of its relationship with taste and imagery of food (Hamdy et al., 1999a). Moreover, the prefrontal cortex has been associated with the perception of body signals, attentional control, and higher order sensorimotor processing (Suntrup et al., 2014). The occipitoparietal regions are regarded as a hub area for integrating sensory input with motor output (Kern M. et al., 2001). Taken together, we speculate that these non-sensorimotor regions could play a supplementary role in the regulation of swallowing linked to swallow-related intent and planning and possibly urges.

In the present study, the dynamic FC displayed a different pattern of brain areas with altered FC compared with the static FC maps. Other studies using EEG with high temporal resolution signals have shown that the activity of the brain is constantly changing over time at rest (Van de Ville et al., 2010). This indicates that dynamic FC analysis might add additional information to our understanding of the mechanism of iTBS on the swallowing cortex. We observed ipsilateral suppression of FC on the left angular area, left thalamus and PCC in the dynamic FC maps in the early windows. Then, increased FC was observed in multiple regions, including the sensorimotor brain network (bilateral primary sensorimotor cortex and paracentral lobule) and non-sensorimotor brain network (frontal, temporal and occipital gyrus), and was seen in the following windows of the dynamic FC maps. We suppose that the decreased FCs in the early stages may be induced by cTBS that yielded an inhibitory after-effect on spontaneous neuron activity, and the following increased FC in multiple brain areas might be caused by iTBS, which produced an excitatory after-effect. Interestingly, in static FC maps, we did not observe an alteration in FC in the multifocal regions including sensorimotor brain areas and non-sensorimotor brain areas, which instead demonstrated increased FC in the dynamic FC maps. This finding indicates that dynamic FC can provide much more information compared with the static FC method for detecting the aftereffects of TBS over the swallowing cortex. Of note, we observed multiple bilateral brain areas with increased FC in the late stages of dynamic FC maps, and the results indicate contralateral iTBS facilitate brain excitability in bilateral swallowing in the motor cortex instead of interhemispheric inhibition.

It is important to consider the limitations of the present study. First, since previous work has shown that sham TBS over the pharyngeal motor cortex does not alter cortical excitability in an unconditioned system (Mistry et al., 2007, 2012), we did not include a sham stimulation group in the present study. Second, the sample size in this study was relatively small, and a larger dataset would be required to validate the present findings. Finally, considering the issue of cooperation of participants during the imaging study, we did not prolong the scanning time to measure the aftereffects of TBS until it returned to baseline.

Taken together, we explored the short-term aftereffects of iTBS following contralateral cTBS (a virtual lesion) on the suprahyoid muscles motor cortex. We observed increased FC in the bilateral sensorimotor cortex and other non-sensorimotor areas induced by contralateral iTBS, which could not be fully displayed in static FC analysis. The present study indicates that dynamic FC analysis can provide much more information about the brain excitability induced by contralateral iTBS. Our results provide evidence that iTBS could be used as a novel noninvasive tool for rehabilitating swallowing difficulties after brain damage. We suggest that contralateral iTBS might be developed as a therapeutic strategy for swallowing disorders associated with unilateral lesions in the swallowing cortex.

The present study was approved by the clinical research ethics committee of Guangzhou First People’s Hospital. The study was conducted in accordance with the Declaration of Helsinki (2008 revision).

## Author Contributions

XW contributed to the experimental design and writing of the manuscript. GZ, XR, and YuLi were involved in the literature review, data collection, and writing of the manuscript. CG, SY, LL, and XC contributed to the analysis of MRI data. LJ, EL, and YaLi were involved in the data collection. GX, XJ, and YLa contributed to the experimental design and the writing process.

## Conflict of Interest Statement

The authors declare that the research was conducted in the absence of any commercial or financial relationships that could be construed as a potential conflict of interest.

## Footnotes

1 http://rfmri.org/DPARSF

2 http://www.fil.ion.ucl.ac.uk/spm

3 http://www.restfmri.net

4 http://afni.nimh.nih.gov/pub/dist/doc/manual/AlphaSim.pdf
